# The Physiologic Cost of Chewing

**Published:** 1919-09

**Authors:** 


### THE PHYSIOLOGIC COST OF CHEWING

The act of mastication has lately attained new prominence in human physiologic routine from two different circumstances: First, the vigorous advocacy of thorough mastication as the basis to hygienic well-being. It is largely owing to the propagandist energies of the late Mr. Horace Fletcher that the doctrine of deliberate mastication has been widely preached; and "Fletcherism" has gained many adherents among that omnipresent group which is searching for the secret of a better existence through improved health. One of the claims made in favor of thorough mastication as advocated by Fletcher is that the extreme comminution of the food and the more effective insalivation promote the digestion and utilization of the nutrients. Despite the plausibility of this argument there is an abundance of experimental evidence to show that the nutrients in the common food products are absorbed in large measure even under ordinary habits of eating. It is exceptional to find less than ninety per cent. of the digestible nutrients utilized; and for the familiar fats and carbohydrates the records approach almost perfection. The residual fecal masses are normally devoid of more than small quantities of digestible nutrients. That which constitutes the output by the bowel comprises indigestible residues, refuse matter from alimentary secretions, and bacterial residues. These are not unutilized nutrients. Furthermore, comparative investigations of the actual utilization of the nutrients by persons who on one occasion ''bolted'' and on another occasion ''Fletcherized'' the same diets have given no experimental justification for the assumption of improved absorption as the effect of the more thorough mastication. Obviously it is a physiologic desideratum to comminute moderately at least some of the meats and carbohydrate foods that enter into the customary human dietary. In certain diseased conditions, comminution of the food is an indispensable requisite. However, so far as the faddist features and the extreme claims of unique benefits are concerned, we can not forego quoting the remark of an extremist in the other direction. ''Fletcherism,'' he writes, ''is permissible in those easy-going lackadaisical individuals whose tastes are gently epicurean and who possess the desires of a Lucullus minus the means. Let those benighted, harmless souls chew and champ to their heart's content, for they, poor beings, need some fad, and this one can harm no one unless it be themselves. But to busy men who are shouldering the cares of government, commerce and science, and whose strenuous impetuosity moves them to act quickly, whose every act is intense and every movement a flash—to such individuals, Fletcherism is a thorn in the flesh.''[1]

---

[1] Fitch, W. E.. Dietotherapy, New York 2: 83, 1918.

In the second place mastication has become conspicuous in this country through the widespread use of chewing gum. Some time ago a distinguished chemist[2] remarked in a public address that if the Rockefeller Institute is spending to good advantage about half a million dollars per annum for medical research, the chewing gum bill of the United States would easily support half a dozen Rockefeller Institutes. Coaxed by the insidious suggestions of advertisements and encouraged by the public example of bankers and ministers, physicians and judges, men and women from all classes have joined the ranks of the mastication army. The lasting odor of mint has begun to compete with that of onions and garlic in certain groups of our population. We shall not venture to discuss the alleged virtues of chewing gum. The reports of its help in allaying thirst for the boys in the trenches can scarcely be used in support of the use of chewing gum in the ordinary walks of life. Nor can the alleged presence of pepsin in certain brands be emphasized as a universal panacea. Amid all the uncertainty as to the physiologic effects of chewing gum one fact has been clearly demonstrated. Benedict and Carpenter[3] of the Nutrition Laboratory of the Carnegie Institution of Washington in Boston have found that as a result of chewing gum the basal metabolism may be increased more than seventeen per cent.

This has a further bearing on the doctrine of "Fletcherizing." If prolonged mastication can necessitate an excess heat production equivalent to nearly one-fifth of the basal metabolism, it is easily seen, to quote the Boston physiologists, that any advantage gained from a possible increase in the digestibility of the food is more than compensated by the increase in heat production. The conception, they

---

[2] Bakeland, L. H.: Some Aspects of Industrial Chemistry, Science, Aug. 7, 1914, p. 197.

[3] Benedict, F. G., and Carpenter, T. M.: Food Ingestion and Energy Transformations with Special Reference to the Stimulating Effects of Nutrients, Carnegie Institution of Washington, Publication 261, 1918.

add, of an increase in the digestibility and in the utilization of the energy of foodstuffs as a result of prolonged mastication thus finds no support in fact.—*Journal A. M. A.*, July 12, 1919.

